# Critical Vertebrobasilar Insufficiency From Left Intracranial Vertebral Artery Stenosis With Contralateral Hypoplasia Presenting as Recurring Vertigo: Urgent Stenting to Prevent the Progression of a Posterior Circulation Stroke

**DOI:** 10.7759/cureus.89981

**Published:** 2025-08-13

**Authors:** Zhuo Luan, Alberto Maud, Xin Yang, Gustavo J Rodriguez

**Affiliations:** 1 Neurology, Texas Tech University Health Sciences Center El Paso, El Paso, USA; 2 Neurology/Interventional Neurology, Texas Tech University Health Sciences Center El Paso, El Paso, USA

**Keywords:** hypoplastic vertebral artery, intracranial stenting, posterior circulation stroke, recurrent vertigo, vertebral artery stenosis, vertebrobasilar insufficiency (vbi)

## Abstract

A 67-year-old man with a history of hypertension and untreated dyslipidemia presented with a four-day history of frequent episodic vertigo associated with nausea and vomiting. The patient experienced up to 20 episodes of spinning vertigo per day, each lasting several minutes, without provocation by head movement. MRI confirmed acute infarcts in the right posterior cerebral and cerebellar territories, and CTA revealed severe stenosis of the left vertebral artery V4 segment, with a hypoplastic right vertebral artery. Given refractory symptoms despite dual antiplatelet therapy and permissive hypertension, urgent intracranial balloon angioplasty and balloon-mounted drug-eluting stent placement was performed. The patient recovered completely without further dizziness or neurological deficit. This case emphasizes that recurrent atypical vertigo and brief syncope may be warning signs of vertebrobasilar insufficiency, especially in the context of vertebral artery stenosis with limited collateral flow. Timely vascular imaging and intervention can prevent subsequent debilitating brainstem stroke.

## Introduction

Vertebrobasilar insufficiency (VBI) is a potentially serious condition that can mimic benign vestibular disorders such as vestibular neuronitis or vestibular migraine [1]. This diagnostic overlap can be particularly challenging in elderly patients, where common symptoms like recurrent vertigo are often misattributed to peripheral vestibular etiologies. However, in individuals with vascular risk factors including hypertension, diabetes, hyperlipidemia, and smoking history, these symptoms should prompt careful evaluation for possible posterior circulation compromise [2].

Isolated vertigo as a manifestation of vertebrobasilar pathology remains under-recognized in clinical practice [3]. This under-recognition contributes to significant diagnostic delays, increasing the risk of major cerebrovascular events such as brainstem or cerebellar infarction [4]. Atherosclerotic disease affecting the intracranial vertebral arteries can significantly impair posterior circulation perfusion, especially in the presence of anatomical variations such as contralateral vertebral artery hypoplasia. These anatomical factors may limit compensatory blood flow and heighten the risk of ischemic events [5].

Red-flag features such as recurrent or prolonged vertigo, especially when accompanied by brainstem symptoms such as diplopia, dysarthria, limb weakness, or transient loss of consciousness should raise suspicion for a central etiology [6]. Prompt neuroimaging and vascular assessment are essential to identify critical stenoses or occlusions. While optimal medical therapy including antiplatelets, statins, and risk factor modification remains the cornerstone of treatment for intracranial atherosclerosis, select patients with symptomatic high-grade stenosis may benefit from endovascular intervention [7].

We present the case of a 67-year-old man with recurrent, atypical vertigo found to have severe stenosis of the left intracranial vertebral artery in the context of a hypoplastic contralateral vertebral artery. Due to the high risk of posterior circulation infarction, he underwent successful endovascular stenting, resulting in complete and sustained resolution of vertigo symptoms. This case highlights the importance of recognizing central causes of vertigo and the potential role of endovascular therapy in appropriately selected patients with vertebrobasilar insufficiency.

## Case presentation

A 67-year-old man with a history of hypertension managed with losartan, untreated dyslipidemia due to statin intolerance, and no known cardiac disease presented to the emergency department with a four‑day history of frequent vertigo episodes, nausea, and vomiting. He reported up to 20 episodes per day, each lasting approximately several minutes, described as spinning sensations. The symptoms were not triggered by changes in head position or body posture. Some features of the vertigo closely mimicked vestibular neuronitis, as the symptoms partially improved with meclizine, although the episodes were not positional and occurred in both lying and standing positions. No nystagmus was observed after the patient arrived at the hospital, likely because the patient had already received meclizine. In addition, he reported transient loss of consciousness lasting a few seconds during vertigo-induced vomiting, a feature that is highly unlikely in peripheral causes of vertigo. He denied any auricular fullness, tinnitus, or hearing loss. Neurologic examination was unremarkable, with no focal deficits and a National Institutes of Health Stroke Scale (NIHSS) score of 0. The patient had no nystagmus; Dix-Hallpike and head impulse tests were negative. There was no dysmetria or ataxia, and gait was normal.

**Figure 1:**
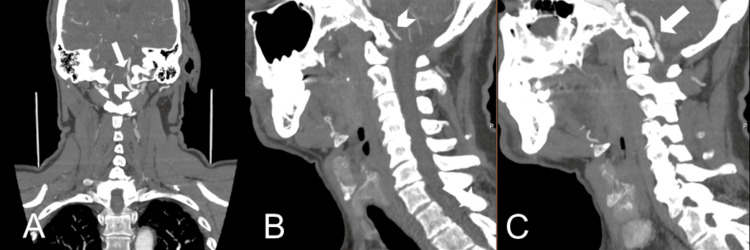
CTA of the head and neck on admission. A: coronal image; B: sagittal image from the right side; C: sagittal image from the left side. A hypoplastic right vertebral artery is seen in panels A and B (arrowheads). High-grade stenosis involving the distal left vertebral artery (intracranial V4 segment) is visualized in panels A and B (arrows).

Initial head CT was negative for acute intracranial changes. CTA revealed high‑grade (80-90%) stenosis of the V4 segment of the left vertebral artery and a hypoplastic right vertebral artery (measuring less than 1 mm in diameter near the vertebrobasilar junction) (Figure 1).

**Figure 2:**
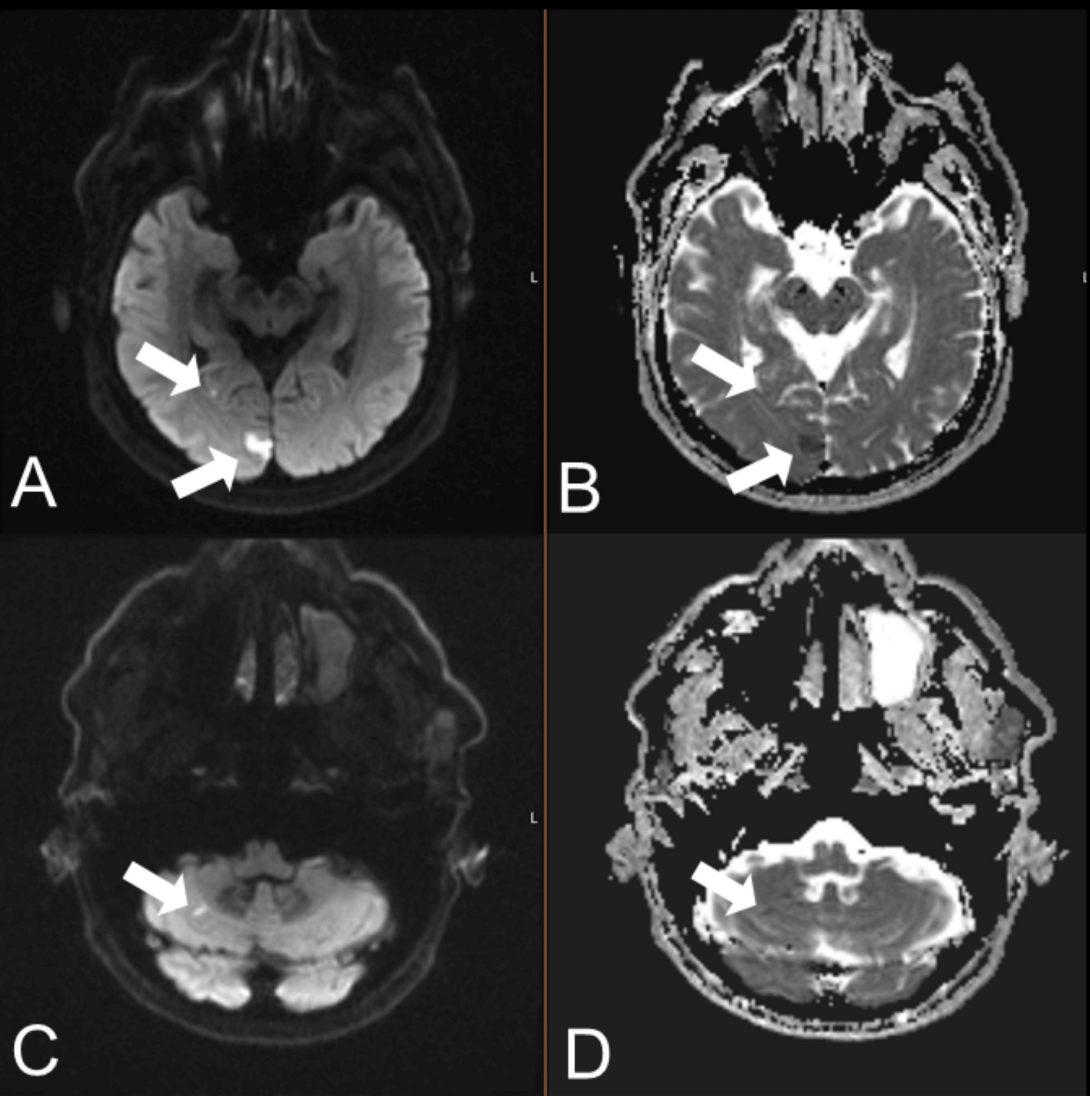
Brain MRI in the axial plane. A and C: DWI sequences; B and D: Corresponding ADC sequences. The images demonstrate multiple small acute ischemic infarcts showing restricted diffusion (hyperintensity on DWI and corresponding low signal on ADC) in the right occipital lobe, right posterior temporal lobe, and right cerebellum (arrows). These infarcts are within the vascular territories of the right posterior cerebral artery and the right superior cerebellar artery. DWI: diffusion-weighted imaging, ADC: apparent diffusion coefficient

MRI confirmed multiple acute infarcts in the right occipital lobe, right posterior temporal lobe, and right cerebellum, in the distribution of the right posterior cerebral and superior cerebellar arteries (Figure 2).

The patient’s lipid profile revealed hypercholesterolemia and hypertriglyceridemia, both of which are associated with an increased risk of cerebrovascular and cardiovascular disease (Table 1).

**Table 1:** Lipid profile The patient’s lipid profile revealed hypercholesterolemia and hypertriglyceridemia, with elevated total cholesterol and low-density lipoprotein (LDL) levels, reduced high-density lipoprotein (HDL), and an increased total cholesterol-to-HDL ratio.

Serum	Value	Reference
Cholesterol	261	50-200 mg/dL
Triglycerides	200	35-150 mg/dL
HDL	30	40-59 mg/dL
LDL	191	50-100 mg/dL
Total/HDL ratio	8.7	1.0-5.0

Given the posterior circulation infarcts and concerning vascular anatomy, the patient was started on aspirin, clopidogrel, and IV fluids. In light of severe stenosis of the left vertebral artery and hypoplasia of the contralateral vessel, permissive hypertension was employed to support basilar artery perfusion. We were unable to initiate statin therapy, as the patient had previously experienced statin intolerance characterized by muscle aches and weakness. His primary care provider had trialed multiple statins in the past without success, and the patient currently declined any further attempts at statin therapy. Because of the critical changes in both vertebral arteries and the high risk of basilar artery ischemic stroke, the neuro‑interventional team was consulted on the day of admission.

**Figure 3:**
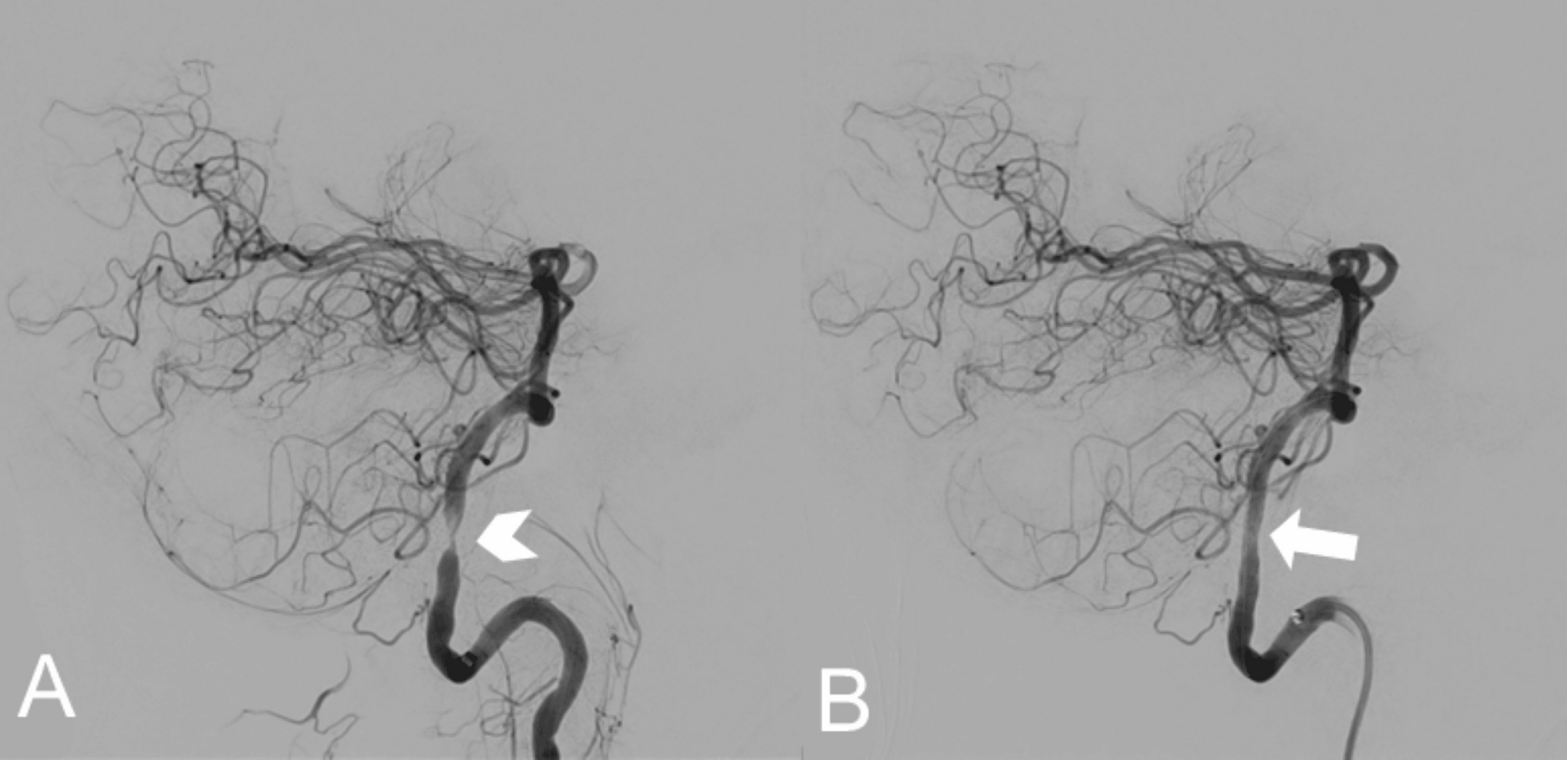
Digital subtraction angiography (DSA) of the left vertebral artery in lateral projection. A: Lateral view demonstrating 80-90% atherosclerotic stenosis (arrowhead) in the V4 segment of the left vertebral artery, with preserved flow in the distal cortical branches. B: Post-procedure lateral view showing no residual stenosis (arrow) following intracranial balloon angioplasty with placement of a drug-eluting balloon-mounted stent. There is no evidence of in-stent thrombosis.

The risks associated with cerebral angiography such as bleeding, infection, stroke, thrombosis, and kidney injury were discussed with the patient, and he understood and agreed to proceed with the procedure. Diagnostic cerebral angiography performed on hospital day 2 confirmed high‑grade left V4 stenosis. A balloon‑mounted drug‑eluting stent (3.5 × 18 mm Onyx Frontier) was successfully deployed at the target arterial (Figure 3A). Post‑procedural angiography demonstrated no residual stenosis and excellent distal flow (Figure 3B).

The patient was admitted to the neuro ICU for close monitoring and remained neurologically stable. His vertigo resolved immediately after the procedure, and he was able to ambulate independently the next day. He was discharged on dual antiplatelet therapy and for outpatient angiographic follow‑up. At the six-month follow-up, the patient had no neurological deficits or recurrent episodes, with a modified Rankin scale (mRS) score of 0.

## Discussion

Recurrent vertigo remains one of the most diagnostically challenging symptoms encountered in clinical neurology [4]. While benign peripheral causes such as benign paroxysmal positional vertigo (BPPV), vestibular neuronitis, and vestibular migraine account for the majority of cases, central causes like vertebrobasilar insufficiency are critical to identify due to their potential for irreversible neurological injury. This is particularly true when vertigo presents atypically characterized by prolonged duration and high frequency and is accompanied by “red-flag” features such as syncope, diplopia, dysarthria, limb weakness, or other signs of brainstem involvement [4,6].

In our case, the patient's vertigo was clearly atypical: the episodes were non-positional, occurred more than 20 times daily, each lasting approximately several minutes, and were associated with a brief syncopal event but response to meclizine. These features, in the context of known vascular risk factors, should immediately raise concern for central causes, particularly posterior circulation ischemia. Alternative diagnoses including arrhythmia and seizure were considered. However, the patient’s ECG and continuous telemetry consistently demonstrated sinus rhythm, and there were no clinical features suggestive of seizure, making these diagnoses less likely. The combination of acute infarcts in posterior cerebral and cerebellar territories, severe left vertebral artery stenosis (high‑grade, 80-90% stenosis), and contralateral vertebral artery hypoplasia (less than 1 mm in diameter near the vertebrobasilar junction) pointed toward a hemodynamic mechanism of vertebrobasilar insufficiency, with critically impaired perfusion to the brainstem and cerebellum.

Clinicians must be trained to recognize red-flag features in vertigo presentations that depart from the classic patterns of peripheral vestibulopathies. Vertigo not triggered by positional change, prolonged in duration, or accompanied by even subtle brainstem signs should prompt urgent neuroimaging preferably with CTA or MRA of the head and neck to evaluate for posterior circulation pathology [6]. Failure to do so may delay diagnosis and treatment, increasing the risk of a disabling or fatal stroke.

Intracranial vertebral artery stenosis carries significant risk, particularly when the contralateral vertebral artery is hypoplastic or occluded [8]. In such anatomical scenarios, compensatory collateral flow is severely limited, and perfusion of the posterior circulation especially the basilar artery and its branches becomes highly dependent on the patency of the dominant vertebral artery. In this precarious hemodynamic state, the drops in perfusion can lead to watershed infarcts in the brainstem and cerebellum [9]. In our patient, the right vertebral artery was hypoplastic and unable to provide sufficient collateral flow in the setting of critical stenosis of the left intracranial (V4 segment) vertebral artery. This placed the entire posterior circulation at high risk for failure. The patient presented with acute-onset vertigo over a four-day period, which may have been precipitated by plaque instability or rupture of atherosclerotic plaque in the intracranial vertebral artery. Rather than typical watershed infarcts from global hypoperfusion, the small, scattered embolic infarcts in the posterior cerebral and cerebellar territories were likely caused by plaque fragments dislodged during atherosclerotic plaque rupture [10].

Although the major clinical trial (Stenting and Aggressive Medical Management for Preventing Recurrent Stroke in Intracranial Stenosis, 2011) and following important trials demonstrated that aggressive medical management is generally superior to intracranial stenting in reducing stroke risk due to an unacceptable high peri-procedural risk, the trial excluded patients with vertebrobasilar insufficiency and severely compromised flow due to bilateral vertebral pathology [7,11-13]. In real-world practice, patients with high-grade intracranial stenosis and inadequate collateral circulation especially those with symptoms refractory to maximal medical therapy may require urgent endovascular intervention. In such select cases, balloon-mounted drug-eluting stents have shown improved outcomes and procedural safety [14]. Endovascular stenting restores vessel patency and reestablishes distal perfusion, preventing extension of infarcts and subsequent basilar artery thrombosis or occlusion. The timing of intervention in such cases is crucial. Delaying revascularization in a patient with progressive or persistent vertebrobasilar insufficiency may lead to adverse outcomes, including infarction of the brainstem, cerebellum, or bilateral occipital lobes. A basilar artery stroke, in particular, carries a high risk of severe disability or death [15]. Thus, in patients presenting with recurrent atypical vertigo and vascular risk factors, especially in the context of known vertebral artery disease, the threshold for urgent vascular imaging and neuro-interventional evaluation should be low.

Our case is particular in that the patient had one intracranial vertebral artery stenosis in the setting of a hypoplastic contralateral vertebral artery, an anatomical combination not previously reported. The most similar case we found was described by Sundar et al., who reported a patient with recurrent presyncopal episodes due to bilateral intracranial vertebral artery stenosis, with complete symptom resolution following left vertebral artery angioplasty and stenting [16]. This further supports the role of endovascular intervention in carefully selected, critically symptomatic patients.

Our case demonstrates that early recognition of red-flag vertigo, prompt imaging, and timely endovascular stenting in a high-risk anatomical setting (i.e., vertebral artery stenosis with contralateral hypoplasia) can result in excellent clinical outcomes. The patient recovered fully, without residual neurological deficits, highlighting the value of a proactive diagnostic and therapeutic approach.

## Conclusions

This case illustrates the importance of considering vertebrobasilar insufficiency in elderly patients presenting with recurrent, non-positional vertigo particularly when accompanied by vascular risk factors and atypical features such as prolonged episodes or transient loss of consciousness. Intracranial vertebral artery stenosis, especially in the setting of contralateral hypoplasia, can significantly compromise posterior circulation perfusion and carries a high risk of a life-threatening infarction. Prompt recognition of central vertigo features, early vascular imaging, and timely intervention such as endovascular stenting rather than relying solely on medical therapy are critical to preventing disabling brainstem strokes. This case underscores the need for heightened clinical vigilance and a low threshold for neurovascular evaluation in patients with bilateral vertebral artery disease, especially when one artery is severely stenosed and the other is hypoplastic or occluded.
